# Bioactivity of food peptides: biological response of rats to bovine milk whey peptides following acute exercise

**DOI:** 10.1080/16546628.2017.1290740

**Published:** 2017-03-07

**Authors:** Carolina Soares Moura, Pablo Christiano Barboza Lollo, Priscila Neder Morato, Eder Muller Risso, Jaime Amaya-Farfan

**Affiliations:** ^a^ Food and Nutrition Department, Protein Resources Laboratory, Faculty of Food Engineering, University of Campinas (UNICAMP), Campinas, Brazil

**Keywords:** Bioactive peptides, whey protein hydrolysate, HSP, immune, glycogen

## Abstract

**Background:** Several physiologically beneficial effects of consuming a whey protein hydrolysate (WPH) have been attributed to the greater availability of bioactive peptides.

**Aims:** The aim was to investigate the effect of four branched-chain amino acid- (BCAA-)containing dipeptides, present in WPH, on immune modulation, stimulation of HSP expression, muscle protein synthesis, glycogen content, satiety signals and the impact of these peptides on the plasma free amino acid profiles.

**Methods:** The animals were divided in groups: control (rest, without gavage), vehicle (water), L-isoleucyl-L-leucine (lle-Leu), L-leucyl-L-isoleucine (Leu-lle), L-valyl-Lleucine (Val-Leu), L-leucyl-L-valine (Leu-Val) and WPH. All animals were submitted to acute exercise, except for control.

**Results:** lle-Leu stimulated immune response, hepatic and muscle glycogen and HSP60 expression, whereas Leu-Val enhanced HSP90 expression. All dipeptides reduced glucagon-like peptide-1 and glucose-dependent insulinotropic polypeptide, no changes were observed on leptin. All peptides inhibited NF-kB expression. The plasma amino acid time-course showed peptide-specific and isomer-specific metabolic features, including increases of the BCAAs.

**Conclusion:** The data indicate that lle-Leu was effective to attenuate immune-suppression exercise-induced, promoted glycogen content and stimulated anti-stress effect (HSP). Furthermore, Leu-Val increased HSP90, p-4EBP1, p-mTOR and p-AMPK expression. The data suggest the involvement of these peptides in various beneficial functions of WPH consumption.

## Introduction

Whey proteins (WPs) are known to be functional for promoting the improvement of several biological responses that include cytoprotective effects mediated by the enhancement of heat shock protein (HSP) expression, promotion of glycogen content, stimulation of muscle protein synthesis, modulation of the immune response and improvement of satiety [1–6]. In particular, the HSPs are a natural endogenous defense system capable of promoting cell resistance and tolerance against a variety of stressors, protecting and repairing damage. HSPs maintain cell integrity and structure promoting cell survival during periods of stress [7].

When hydrolyzed, WPs become rich sources of bioactive peptides, including branched-chain amino acid- (BCAA)-containing dipeptides [8], which can act as cell-signaling molecules capable of modulating physiologic functions reaching cells and tissues [9].

The positive health effects of WP consumption, and particularly the whey protein hydrolysate (WPH), have been suggested to be due to the greater availability of bioactive peptides, indicating that one or a combination of several peptides present in the whey could be responsible for the various WPH effects [2].

Several of the positive effects attributed to WP consumption are often associated with exercise and healthy homeostasis. Since exercise itself is known to induce changes in homeostasis, it would be worthwhile from the standpoint of nutrition to deepen our understanding of how these proteins can counteract some adverse changes caused by, for example, raised body temperature, immune suppression, muscle damage and fast glycogen depletion, all such alterations likely to occur during or after acute exercise.

Hypothesis: given that dipeptides L-leucyl-isoleucine (Leu-lle), L-isoleucyl-leucine (lle-Leu), L-valyl-leucine (Val-Leu) and L-leucyl-valine (Leu-Val) are dipeptides present in the milk whey proteins, and that they are sufficiently stable to withstand isolation from a hydrolysate, we then hypothesized that any one of these dipeptides could display bioactivity explaining some of the metabolic effects attributed to the hydrolyzed milk whey proteins. Thus, the purpose of the present study was to investigate the effect of four BCAA-containing dipeptides, present in WPH, on immune modulation, stimulation of HSP expression, muscle protein synthesis, glycogen content and satiety signals. Owing to the dual role of BCAAs and their peptides as indispensable nutrients and cell possible signalers, it should be also instructive to monitor how the plasma free-amino-acid profiles respond to the intake of these four dipeptides. Therefore, the results could indicate which peptides (whey components) could be involved or be responsible for several effects attributed to WPH consumption.

## Materials and methods

### Ethical approval

All animal manipulations were performed according to the institutional guidelines of the Ethics Commission on Animal use of the University of Campinas (UNICAMP, Brazil) that approved full experimental procedures (CEUA-UNICAMP, protocol number 2845–1).

### Animals and experimental trials

For the first experiment, 56 male Wistar rats (21 days old, specific-pathogen free) from the Multidisciplinary Center for Biological Research (University of Campinas, SP, Brazil) were housed in individual cages with access to chow (Labina, Purina, Brazil) and water ad libitum, and maintained under controlled ambient conditions (reverse 12-hour light/dark cycle, 55% humidity, 22 ± 1°C). After growth, the animals (7 weeks of age and 278 g ± 14.33 of body weight) were divided into seven groups (n = 8): control (rest, no gavage), vehicle (water), L-isoleucyl-L-leucine (lle-Leu), L-leucyl-L-isoleucine (Leu-lle), L-valyl-L-leucine (Val-Leu), L-leucyl-L-valine (Leu-Val) and whey protein hydrolysate (WPH). These BCAA-containing dipeptides have been identified from WPH [8]. The choice of these peptides was based on previous results showing positive effects of some of them [10]. Additionally, it has been shown that peptides containing branched chain amino acids can reach the circulation and tissues in their intact form and thus pass through the gastrointestinal tract without any modification [11]. All groups were submitted to a single exercise bout on a treadmill without inclination at the speed of 18 m/min for 60 min, except for control [12]. Immediately after the exercise, the animals were gavaged with the dipeptides, WPH or water (vehicle) and were sacrificed 3 hours after gavage. The immune system, heat-shock protein (HSP) expression, satiety and glycogenic responses were investigated.

In the second experiment, 60 animals with the same characteristics, same feeding treatment, same dose and same exercising protocol, as defined above, were used to time-course curve (0, 15, 30, 45, 60 min). Immediately after the exercise, the animals were gavaged and a 60-min time-course of plasma amino acids was performed. Here, the objective was to observe the impact of each dipeptide on the plasma amino-acid profiles. For this purpose, the animals were divided into six groups: vehicle (control), lle-Leu, Leu-lle, Val-Leu, Leu-Val and WPH.

### Peptides and whey protein hydrolysate

Each animal received single dose a 3 mmol (0.75 g/kg) of the peptides or WPH (0.75 g/kg) dissolved in water by oral gavage [13]. All dipeptides (purity > 98%) were produced by BioBasic (Markham, Ontario, Canada). The WPH was from Hilmar Ingredients (Hilmar, CA, USA).

### Blood collection and biochemical parameters

Three hours after peptides gavage, blood samples were collected and centrifuged at 3000 × g (4°C, 15 min) to obtain the serum. All kits were used following manufacturer protocol and were purchased from Millipore: glucose-dependent insulinotropic polypeptide (GIP), glucagon-like peptide-1 (GLP-1) and C-peptide (Milliplex, RMHMAG-84K), insulin levels (ELISA, EZRMI-13K), interleucine 1β and interleucine 10 (Recytmag-65K) and leptin (Radpk-81K). For hematological parameters (immune system) the blood was collected with EDTA anticoagulant and analysed using an automated cell counter (Ac.T5diff hematology analyzer, Beckman Coulter, High Wycombe, UK).

### Western blotting procedure

Soleus muscle sample was removed from the animals and immediately frozen in liquid nitrogen for subsequent analysis. Muscle samples were homogenized in antiprotease buffer according to a previously described method [2]. Protein content of muscle homogenate was measured by the Lowry method [14]. The samples were separated by SDS-PAGE and transferred to a nitrocellulose membrane (Santa Cruz, 0.22 μm pore size) using a semi-dry (Bio-Rad, CA, USA). The blots were incubated with the appropriate antibodies: Enzo Life Sciences – HSP90 (ADI-SPA 831), HSP60 (ADI-SPA 806), GAPDH (ADI 905734); Abcam: OGT (ab59135), NF-kB p65 (Ab7970), BCKDH (ab59747), p-AMPK alpha 1 + alpha 2 (ab39400), AMPK (ab80039), IDE (ab25733); Cell Signaling Technology: p-mTOR Ser 2448 (2971S), mTOR (MA 2972), p-4EBP1 T37/46 (94595); Santa Cruz: Akt (H-136) (sc8312), p-AKT Ser 473 (sc7985-R); Bethyl: 4EBP1 (TX A300501A); Upstate Biotechnology: PI3-Kinase (p85), N-SH2 domain (06–496). The membranes were visualized with a UVITEC Cambridge instrument (model Alliance LD2). The quantification of blots was performed with the digital program Image J.

### Plasma time-course free amino acids

Blood samples were collected from the tail vein for each rat at different times with heparin anticoagulant to time-course of 0, 15, 30, 45, 60 minutes after peptides gavage and centrifuged at 3000 × g (4°C, 15 min) to obtain plasma and stored at −20°C. The free amino acids from plasma (100 mg) were extracted with a solution composed in methanol (≥ 99.9%, HPLC grade) and 0.1 M hydrochloric acid (80/20, v/v), and made molecular exclusion filtration (Vivaspin 500 Sartorius). Extract (40 μL) was evaporated in a vacuum cryogenic system. The evaporated was redissolved and homogenized in 20 μL (methanol, triethylamine and ultrapure water, ≥ 99.9%, HPLC grade + PITC phenylisothiocyanate) for derivatization at room temperature and evaporated again. The derivatized samples were dissolved in dibasic anhydrous sodium phosphate buffer (pH 7.4) and chromatographed (Agilent Technologies 1200 series) using a Luna C-18, 100 Ǻ; 3 µ, 250 × 4.6 mm (00G-4251-E0) column at 46°C, detected at 254 nm [15].

### Glycogen content

The liver, heart, kidney and muscle tissues were used for glycogen determination. Tissues samples were digested with potassium hydroxide until totally dissolved and the glycogen precipitated with ethanol. The precipitated glycogen was reacted with phenol–sulphuric acid method, as previously described [16]. The absorbance was read in a spectrophotometer (Beckman-Coulter DU 640) at 490 nm.

### Statistical analysis

Data were presented as mean and standard error (mean ± SEM) and were analysed by ANOVA, followed by the Duncan post-hoc test, using SPSS (Statistical Package for the Social Sciences, Chicago, USA – software version 17.0). The level of statistical significance was set at p < 0.05. All graphs were performed using GraphPad Prism (CA, USA).

## Results

### Protein expression

**Figure 1.**
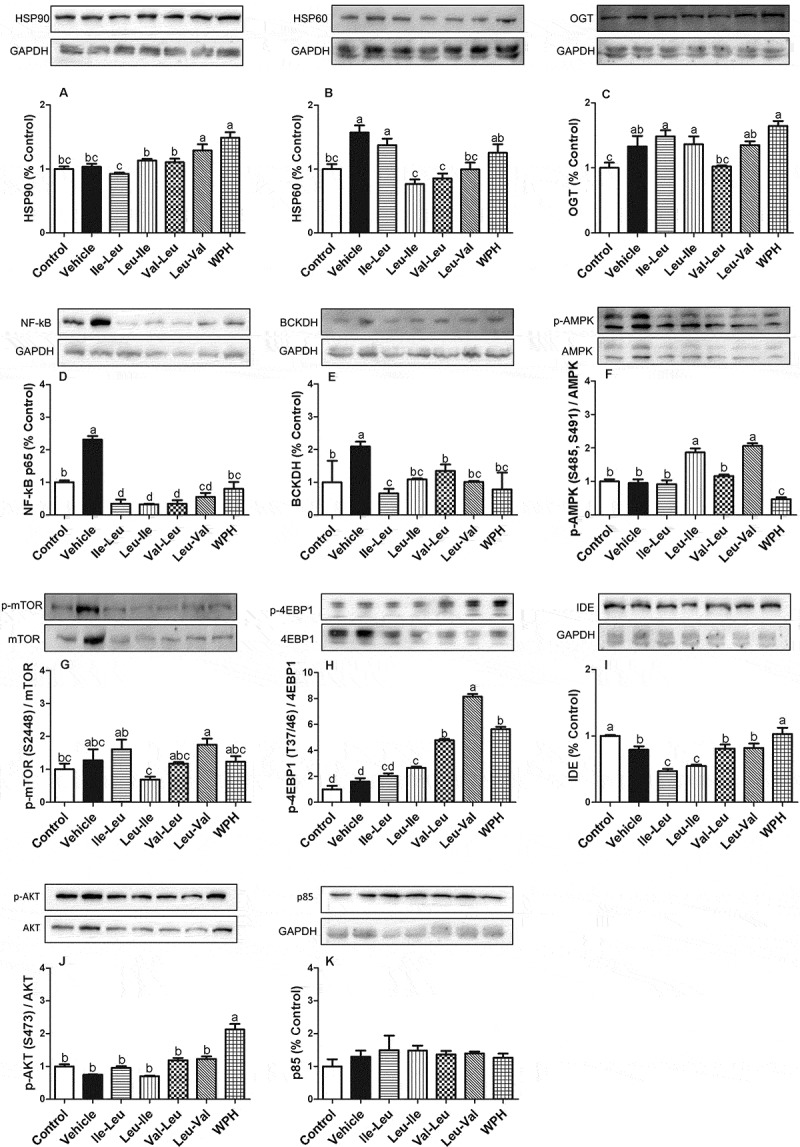
Means (± SEM) of the Western blot analysis (n = 8): HSP90 (a), HSP60 (b), OGT (c), NF-kB p65 (d), BCKDH (e), p-AMPK (f), p-mTOR (g), p-4EBP1 (h), IDE (i), p-AKT (j), p85 (k). Different letters represent significant differences (p < 0.05). GAPDH is loading control. Groups: control (rest, without gavage), vehicle (water), L-isoleucyl-leucine (lle-Leu), L-leucyl-isoleucine (Leu-lle), L-valyl-leucine (Val-Leu), L-leucyl-valine (Leu-Val) and whey protein hydrolysate (WPH).

Of the WPH peptides, Leu-Val increased HSP90 expression, whereas lle-Leu elevated HSP60 in comparison to the other peptides (Figures 1(a, b)). The expression of O-β-acetylglucosaminyltransferase (OGT) was high in all peptides, with exception of Val-Leu, which did not differ from the control (Figure 1(c)). All peptides reduced the muscle nuclear factor kappa B (NF-kB) expression (Figure 1(d)).

Exercise increased the branched-chain α-keto acid dehydrogenase (BCKDH) expression (control versus vehicle) and Val-Leu peptide was elevated in comparison with lle-Leu, but remained at control level (Figure 1(e)). Leu-lle and Leu-Val peptides stimulated the 5ʹ-AMP-activated protein kinase (AMPK) phosphorylation (Figure 1(f)). Leu-Val stimulated the mTOR fosforilation (Figure 1(g)) and 4EBP1 expression (Figure 1(h)). Insulin-degrading enzyme (IDE) was reduced in all peptides in comparison with control (Figure 1(i)). We did not find differences in AKT and p85 responses (Figure (j,k) respectively).

### Glycogen content and blood parameters

**Figure 2.**
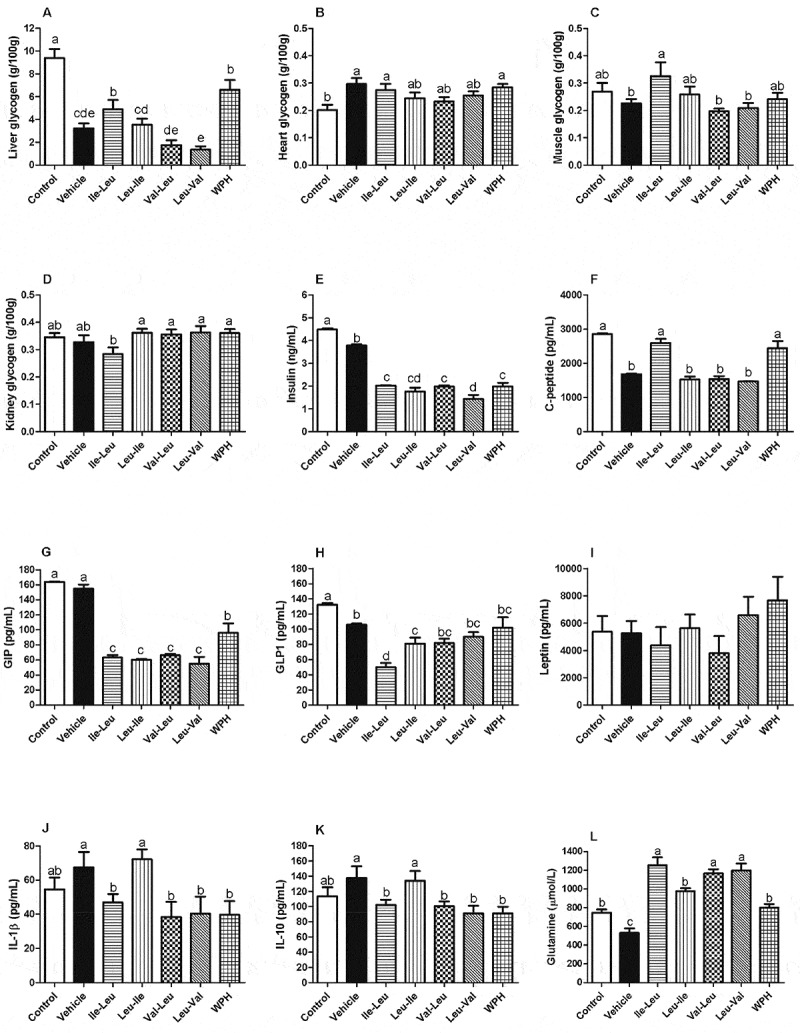
Means (± SEM) for glycogen content: (a) liver (b) heart (c) muscle (d) kidney. Means (± SEM) for serum biochemical parameters: (e) insulin, (f) C-peptide, (g) GIP, (h) GLP-1, (i) leptin, (j) IL-1β, (k) IL-10, (l) free glutamine (μmol/L). Different letters represent significant differences (*p < *0.05). Groups: Control (rest, without gavage), vehicle (water), L-isoleucyl-leucine (lle-Leu), L-leucyl-isoleucine (Leu-lle), L-valyl-leucine (Val-Leu), L-leucyl-valine (Leu-Val) and whey protein hydrolysate (WPH).

Exercise reduced liver glycogen and lle-Leu was the dipeptide that better preserved glycogen content in liver compared to other dipeptides (Figure 2(a)). lle-Leu enhanced the exercise-induced heart glycogen content in comparison with control, but there was no difference between the dipeptides (Figure 2(b)). Again, lle-Leu promoted high accumulation of muscle glycogen compared to the vehicle, Val-Leu and Leu-Val dipeptides (Figure 2(c)). In contrast, lle-leu dipeptide showed the lowest content of kidney glycogen compared to other peptides (Figure 2(d)), but remained at control levels.

All peptides reduced levels of insulin compared to the control (Figure 2(e)). The exercise reduced C-peptide levels for all peptides, with the exception of the lle-Leu and WPH, compared to control (Figure 2(f)). All peptides and WPH reduced glucose-dependent insulinotropic polypeptide (GIP) levels, but there was no difference between the dipeptides (Figure 2(g)). For the glucagon-like peptide-1 (GLP-1), the lle-Leu group showed the lowest level, compared to the other peptides (Figure 2(h)). No change was found in leptin levels (Figure 2(i)). The levels of both interleukin (IL-1β and IL-10) tended to increase with Leu-lle in comparison with other peptides, however it did not differ from control (Figures 2(j, k)). The lle-Leu, Val-Leu and Leu-Val peptides stimulated plasma glutamine levels (Figure 2(l)).

### Immune system

The data in Table 1 show that the one bout of acute exercise affected the immune system, reducing lymphocytes, monocytes, platelet, erythrocytes, average cell volume (MCV) and increasing mean corpuscular hemoglobin concentration (MCHC), leukocytes and neutrophils (comparison of control (at rest) with vehicle (exercised). The lle-Leu stimulated both leukocytes and neutrophils, while Val-Leu increased only neutrophils. On the other hand, no peptide, except for lle-Leu was able to avert the reduction of lymphocytes caused by exercise, only lle-Leu was the peptide that better preserved lymphocytes levels, however it did not restore it to control level. Leu-lle increased monocytes and platelet. No alterations were observed in eosinophils, basophils and hematocrit (Hct). Leu-Val elevated erythrocytes and hemoglobin levels.

**Table 1.** Immune system.

	Erythrogram					Leukogram						
	Erythrocytes	Hct	Hemoglobin	MCV	MCHC	Leukocytes	Neutrophils	Lymphocytes	Monocytes	Eosinophils	Basophils	Platelet
	(μL·10^3^)	(%)	(g/dL)	(fL)	(g/dL)	(μL)	(μL)	(μL)	(μL)	(μL)	(μL)	(μL·10^3^)
**Control**	7945^c^ (5)	40 (1)	16.05^c^ (0.05)	50.3^a^ (0.01)	40.1^b^ (0.1)	5850^c^ (50)	819^e^ (7)	4914^a^ (42)	58.5^b^ (2.5)	0 (0)	0 (0)	1.023^b^ (6)
**Vehicle**	7585^d^ (15)	37 (1)	15.95^c^ (0.04)	48.7^b^ (0.15)	43.05^a^ (0.20)	6200^b^ (100)	2480^a^ (40)	3720^d^ (60)	0^d^ (0)	0 (0)	0 (0)	967^c^ (8)
**lle-Leu**	7905^c^ (25)	39 (1)	16.45^b^ (0.05)	49.25^b^ (0.10)	42.15^a^ (0.15)	6700^a^ (100)	2412^a^ (36)	4221^b^ (63)	67^b^ (1)	0 (0)	0 (0)	1016^b^ (10.5)
**Leu-lle**	7879^c^ (5)	38 (1)	15.95^c^ (0.07)	48.15^b^ (0.05)	41.95^a^ (0.12)	5550^d^ (50)	1498^d^ (13)	3885^c^ (35)	111^a^ (1)	0 (0)	0 (0)	1062^a^ (8.5)
**Val-Leu**	7885^c^ (5)	38 (1)	16.15^c^ (0.04)	48.15^b^ (0.05)	42.45^a^ (0.1)	5350^d^ (50)	2461^a^ (23)	2782^e^ (26)	53.5^b^ (3.0)	0 (0)	0 (0)	938^d^ (2)
**Leu-Val**	8565^a^ (25)	39 (1)	17.05^a^ (0.05)	45.5^d^ (0.1)	43.65^a^ (0.25)	4300^e^ (10)	1720^c^ (3)	2537^f^ (10)	43^c^ (0.3)	0 (0)	0 (0)	983^c^ (6)
**WPH**	8415^b^ (25)	39 (1)	16.8^a^ (0.03)	46.3^c^ (0.13)	43^a^ (0.1)	6050^bc^ (50)	2299^b^ (19)	3690^d^ (30)	60^b^ (0.5)	0 (0)	0 (0)	985^c^ (9)

Mean and (SEM). Different letters in the same column represent significance differences (p < 0.05). Hct: hematocrit; MCV: mean cell volume; MCHC: mean corpuscular hemoglobin concentration.

### Plasma free amino acids time-course

**Figure 3.**
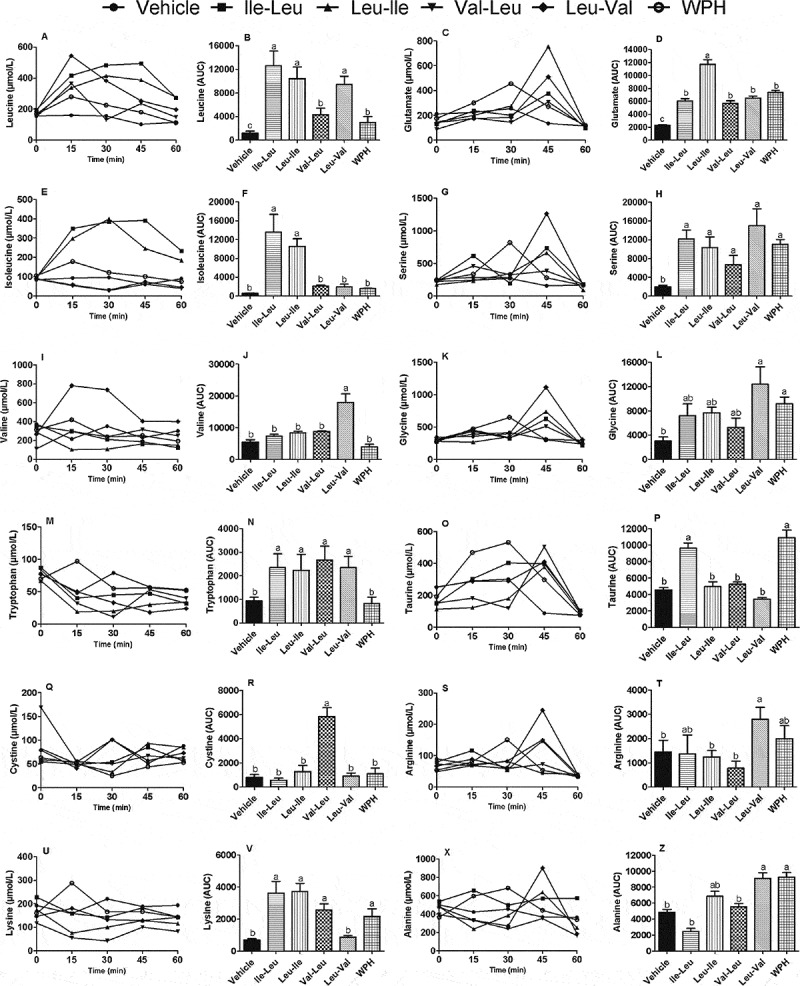
Plasma free amino acids profile (μmol/L) showing the time-course: (a) plasma leucine, (b) Leucine area under the curve (AUC), (c) plasma glutamate levels, (d) Glutamate AUC, (e), plasma isoleucine levels, (f) Isoleucine AUC, (g) plasma serine, (h) Serine AUC, plasma valine (i), Valine AUC (j), glycine levels (k), Glycine AUC (l), plasma tryptophan (m), tryptophan AUC (n), plasma taurine (o), Taurine AUC (p), plasma cystine (q), cystine AUC (r), plasma arginine (s), arginine AUC (t), plasma lysine levels (u), lysine AUC (v), plasma alanine (x), Alanine AUC (z). Different letters represent significant differences (p < 0.05). Groups: vehicle (water), L-isoleucyl-leucine (lle-Leu), L-leucyl-isoleucine (Leu-lle), L-valyl-leucine (Val-Leu), L-leucyl-valine (Leu-Val) and whey protein hydrolysate (WPH). The time-course was done up to 60 minutes because the values of amino acids mostly returned to baseline levels. The vehicle (water) group for this analysis was used as control.

In general, we can observe that the free-amino acid profiles in plasma responded to all peptides (Figure 3). Since all the tested peptides contained only BCAAs, the most relevant results should be the levels of the leucine, isoleucine and valine. Figures 3(a, b) show that all peptides elevated leucine levels, however Val-Leu was in minor proportion. Both lle-Leu and Leu-lle peptides elevated isoleucina levels, and Leu-Val increased valine levels, but Val-Leu did not. With regard to the other amino acids, the data showed that each peptide influenced the outcome in a different and independent fashion, thus producing the elevation of glutamic, serine, cysteine, taurine, lysine, tryptophan, glycine and alanine, but in unique ways.

## Discussion

The purpose of the present study was to investigate if four dipeptides likely to be formed during digestion of the milk WPs could be responsible for some of the metabolic effects attributed to the whey proteins in the rat.

In the normally functional animal body, HSPs are known to play a vital cytoprotective role acting as part of an endogenous defense system, having the mission of repairing damages caused by stressors [7,17]. Increases in the expression of HSPs, therefore, is an adaptive mechanism geared to protect the tissues against drastic changes in homeostasis (anti-stress effect), as occurs during exercise. Supplemental lipoic acid, for instance, was seen to cause an increase in the expression of HSP90, which was associated with a rise in protection to the skeletal muscle of horses exercised on a treadmill [18]. In rodents, treadmill exercise has been successfully used to induce HSP response and alter in homeostasis [1,2,19].

Consistent with this view, we have shown that, in the rat, consuming WPH enhances the muscle expression of exercise-induced HSP90, but without affecting HSP60 [3]. Our present results now indicate that dipeptide Leu-Val may be involved in the WPH capacity to induce HSP90 expression, while only lle-Leu may enhance the exercise-induced HSP60 expression.

O-β-acetylglucosaminyltransferase (OGT) is a protein of the hexosamine pathway that is involved in the enhancement of HSP mediated by glutamine [20]. However, our data did not indicate a relationship between HSP elevation and OGT expression after the consumption of the BCAA-containing dipeptides, similarly to previous results with WPH [2]. Probably a relationship is valid only for glutamine.

It is known that exercise may cause transient changes in immune function by causing immuno-suppression during the first few hours that follow, but generally returning to basal levels in 15 hours. This phenomenon, known as ‘open window’, marks a period when individuals may be more susceptible to infections [21]. The results of the present study (Table 1) support prior evidences that a single exercise bout increases leukocytes and neutrophils [6,22], reduces lymphocyte [23] and apparently does not influence basophils and eosinophils [22]. Additionally, our data showed that platelets were reduced after the exercise, similar result to the one already reported [6].

Several studies have reported that whey protein enhances immune response [5,24–27], but only a few explore the bioactivity of peptides. Peptides Tyr-Gly-Gly and Tyr-Gly, isolated from milk protein were found to have immune modulatory capacity, but the mechanism by which they exert their action remains to be elucidated [9]. Additionally, it has been proposed that BCAAs may also influence immune markers [23]. The present results showed that lle-Leu elevated leukocytes and neutrophils and preserved the lymphocytes levels, indicating that an exercise-induced immune-suppression could be attenuated by lle-Leu dipeptide.

It has been proposed that the high content of glutamine present in WP could somehow be related to its immune modulatory capacity [6,27]. De Moura et al. [1] showed that WPH stimulates glutamine synthetase expression, an enzyme capable of harnessing the nitrogen donated by BCAAs to generate glutamine. Therefore, we determined the concentration of free glutamine in plasma and the results demonstrated that lle-Leu peptide that influenced various immune markers also induced greater free glutamine plasma concentration.

Additionally, once HSPs are produced, they may activate the immune system response [28], however the mechanisms are poorly comprehended. HSPs that seem to influence the immune response are located in extracellular environments or bound to a membrane, and only a few methods exist to identify HSPs in these locations [29]. It is also speculated that HSPs may act in a pro or anti-inflammatory way, depending on the HSP family and type of immune cell in which the HSP interacts [30]. At present, therefore, it is not possible to draw any link between our results and this additional form of activation because our analytical procedure could not distinguish intra from extra-cellular HSPs.

We found only a few studies addressing the effect of whey protein or bioactive peptides on interleukin levels after the stress caused by intense exercise. In humans, Kerasioti et al. (31) showed a tendency to increase anti-inflammatory IL-10, after intense exercise and supplementation with whey. Leu-lle peptide at the same time elevated both pro and anti-inflammatory interleukins, however without increasing muscle NF-kB expression. This discrepancy may be related to the different locations where the parameters interleukins (plasma) and NF-kB (muscle) were analyzed. All peptides reduced the expression in NF-kB, suggesting a protection against the inflammatory process.

It is known that the consumption of WP elevates, recovers and/or preserves the glycogen content in various tissues, even after the acute exercise (3,2). It was recently showed that the intake of lle-Leu and Leu-lle present in WPH did not stimulate liver, muscle or heart glycogen content (10). In contrast, our results indicate that lle-Leu could be involved in the capacity of whey to preserve glycogen (Figure 2). This lack of agreement between the two studies was most likely due to the differences in the time elapsed after consuming the peptides. In the study of Morato et al. (10), the recovery period after gavage was only 30 min, while in the present study was 3 hours. It is believed that kidney glycogen is important for cell survival during oxygen reduced flow periods (32). Our results are consistent with the concept that exercises may provoke a small alteration in kidney glycogen, observed only after 24 hours (33).

It has been proposed that the increase of muscle glucose uptake exercise-induced can proceed through the activation of AMPK (34). A previous study showed that Leu-lle activated AMPK phosphorylation, but with no alteration of the glycogen content (10), consistent with our results. Now we show that the Leu-Val dipeptide also stimulates AMPK phosphorylation, also with no effect on the glycogen.

Whey protein consumption promotes higher satiety compared to other protein sources or to glucose (4,35–37). Previous studies support that whey protein stimulates the secretion of GLP-1 and GIP, both involved in regulating appetite and promoting satiety (36–39). It has also been suggested that peptides present in whey improve satiety signals, but no identification of such peptides or the underlying mechanisms involved have been advanced (40). Our results suggest that none of the BCAA-containing dipeptides are potential contributors to the alleged satiety effect of whey if we considered the effect on GIP and GLP-1. Futhermore, in the present study, the sample collection for analysis of the GIP and GLP-1 parameters were made 3 hours after dipeptide gavage and generally the studies related to satiety show results with shorter time. In addition, the hormonal alterations that regulate the hunger sensation generally are temporary and observed during or soon after exercise, causing a short-term reduction of appetite (41–43). The reduction of GIP and GLP-1 levels found after the exercise (Figures 2(g, h)), seem to be coherent. Once the exercise depletes energy and promotes a predominant catabolic state, while the rested animal (control) spends less energy, leading to an extended satiety effect.

C-peptide and insulin are equally stocked and secreted in blood flow in equimolar quantity (44). Since the C-peptide has a half-life approximately 10 times longer than insulin (45), this may explain why we found different levels for each in our experiment. C-peptide was found to have biological activity as it was demonstrated to produce an increase in skeletal muscle blood flow when administrated to type-1 diabetes patients (44). It was recently reported that the whey consumption stimulates reduction of glycemia through insulin-independent mechanisms because it concomitantly reduces the levels of insulin and C-peptide (38), consistent with our results that showed reduced levels of insulin and C-peptide by all peptides, except for lle-Leu, with respect to C-peptide levels.

It appears that little is known about the factors governing IDE expression, including whether whey protein-derived peptides could have any influence. It is known that IDE is involved in insulin degradation. Kim et al. (46) found that IDE was overexpressed in swimmers after four weeks, thus suggesting that the reduction on insulin levels caused by the exercise could be more related with the increase in clearance than with changes in insulin liberation. However, in the present study, we did not observe IDE overexpression after a single exercise bout (Figure 1(i)).

Regarding the muscle protein synthesis biomarkers, mTOR and 4EBP1, WP consumption is known to activate this pathway, especially if in association with training. Lollo et al. (47) showed that supplementation with leucine activated the mTOR pathway, independent of the protein source (whey or casein) consumed. Our results suggested that Leu-Val peptide could have contributed to the overall effect of whey because the peptide elevated both mTOR and 4EBP1, in spite of not having reached the stage of any muscle mass increase (data not shown) in one single exercise bout.

The plasma amino acids did respond rather specifically to some of the peptides thus suggesting that metabolic adjustments occur, whether through their cell-signaling properties or by the mere presence of the component amino acids once released by hydrolysis. One interesting feature was the increase that a peptide containing only BCAAs can produce of amino acids other than BCAAs, which could be the result of either a specific imbalance created in the amino acid pool or by a peptide’s remote action. For instance, peptides lle-Leu increased taurine, whereas Val-Leu increased cysteine (Figure 3). In this case, the increases of cysteine and taurine can be associated with muscle function (48).

Both lle-Leu and Leu-lle underwent hydrolysis and released their constituent amino acids showing no isomer-specific differences between each other. However, in the synthesis of cysteine (Figure 3(r)) there was a big difference between isomers Val-Leu and Leu-Val, suggesting a complementary action in the two-step production of arginine (Figures 3(r, t)).

Several other features worthy of mention were: 1) the late release (peaking at 45 min) of Glu, Ser, Cys, Tau, Arg and Ala, suggested the need of some time for their syntheses to be accomplished; 2) the trend of WPH to substantially contribute to the production of Glu, Ser, Gly, Tau, Arg and Ala, which was suggestive of other peptide sequences with additional functions, 3) despite the variations found in the profile of the free amino acids (time-course), there was no stimulation in BCKDH expression, (responsible for BCAA catabolism) (49) after the consumption of dipeptides. One limitation of this study is that although the four peptides have been proven to occur in whey protein sequences, their presence in the blood was not determined. Previous results have shown that lle-Pro-Pro milk dripeptide sequence was absorbed intact into the circulation in humans (11). From our data, however, it can be inferred that if present in the blood, these peptides produce significant alterations in the free amino acid profile, suggestive of their permanence and degradation over time.

## Conclusion

We have shown that four BCAA-containing peptides present in WPH can modulate important biological parameters following acute exercise. The results indicate that lle-Leu and Leu-Val dipeptides stood out for displaying functions that have been attributed to the WPH. lle-Leu dipeptide could be involved in the enhancement of glycogen, provide anti-stress effect (HSP) and attenuate exercise-induced immunosuppression. Finally, Leu-Val dipeptide enhanced HSP90, mTOR and 4EBP1 expressions. Functional features emerged relevant to how the very presence of each peptide can affect metabolism, suggesting that a series of commands issued by multiple combinations of peptides could come into play to grant the body reduced stress, higher immune capacity, eased protein synthesis and tissue protection.
